# Gastrointestinal Degradation of Fumonisin B_1_ by Carboxylesterase FumD Prevents Fumonisin Induced Alteration of Sphingolipid Metabolism in Turkey and Swine

**DOI:** 10.3390/toxins8030084

**Published:** 2016-03-21

**Authors:** Sabine Masching, Karin Naehrer, Heidi-Elisabeth Schwartz-Zimmermann, Mihai Sărăndan, Simone Schaumberger, Ilse Dohnal, Veronika Nagl, Dian Schatzmayr

**Affiliations:** 1 BIOMIN Holding GmbH, Erber Campus 1, 3131 Getzersdorf, Austria; sabine.masching@biomin.net (S.M.); karin.naehrer@biomin.net (K.N.); simone.schaumberger@biomin.net (S.S.); 2 Christian Doppler Laboratory for Mycotoxin Metabolism, Center for Analytical Chemistry, Department for Agrobiotechnology (IFA-Tulln), University of Natural Resources and Life Sciences, Vienna (BOKU), Konrad Lorenz Str. 20, 3430 Tulln, Austria; 3 Faculty of Veterinary Medicine Timișoara, Calea Aradului 119, 300645 Timișoara, Romania; mihai.sarandan@gmail.com; 4 BIOMIN Research Center, Technopark 1, 3430 Tulln, Austria; ilse.dohnal@biomin.net (I.D.); veronika.nagl@biomin.net (V.N.); dian.schatzmayr@biomin.net (D.S.)

**Keywords:** detoxification, biotransformation, feed additive, *Fusarium* toxins, biomarker, metabolism, toxicokinetics, swine, poultry

## Abstract

The mycotoxin fumonisin B_1_ (FB_1_) is a frequent contaminant of feed and causes various adverse health effects in domestic animals. Hence, effective strategies are needed to prevent the impact of fumonisins on livestock productivity. Here we evaluated the capability of the fumonisin carboxylesterase FumD to degrade FB_1_ to its less toxic metabolite hydrolyzed FB_1_ (HFB_1_) in the gastrointestinal tract of turkeys and pigs. First, an *ex vivo* pig model was used to examine the activity of FumD under digestive conditions. Within 2 h of incubation with FumD, FB_1_ was completely degraded to HFB_1_ in the duodenum and jejunum, respectively. To test the efficacy of the commercial application of FumD (FUM*zyme*) *in vivo*, female turkeys (*n* = 5) received either basal feed (CON), fumonisin-contaminated feed (15 mg/kg FB_1_+FB_2_; FB) or fumonisin-contaminated feed supplemented with FUM*zyme* (15 U/kg; FB+FUM*zyme*) for 14 days *ad libitum*. Addition of FUM*zyme* resulted in significantly decreased levels of FB_1_ in excreta, whereas HFB_1_ concentrations were significantly increased. Compared to the FB group (0.24 ± 0.02), the mean serum sphinganine-to-sphingosine (Sa/So) ratio was significantly reduced in the FB+FUM*zyme* group (0.19 ± 0.02), thus resembling values of the CON group (0.16 ± 0.02). Similarly, exposure of piglets (*n* = 10) to 2 mg/kg FB_1_+FB_2_ for 42 days caused significantly elevated serum Sa/So ratios (0.39 ± 0.15) compared to the CON group (0.14 ± 0.01). Supplementation with FUM*zyme* (60 U/kg) resulted in gastrointestinal degradation of FB_1_ and unaffected Sa/So ratios (0.16 ± 0.02). Thus, the carboxylesterase FumD represents an effective strategy to detoxify FB_1_ in the digestive tract of turkeys and pigs.

## 1. Introduction

Fumonisins are a group of mycotoxins mainly produced by *Fusarium verticillioides* and *F. proliferatum*. Numerous fumonisin analogues have been identified so far, among them fumonisin B_1_ (FB_1_), fumonisin B2 (FB_2_), and fumonisin B3 (FB_3_) [1]. Based on both prevalence and toxicity, FB_1_ is clearly the most relevant [2]. The major agricultural commodity affected by fumonisins is maize [3]. Recent surveys highlight the widespread prevalence of FB_1_ (56%) in feed and feed raw materials, with concentrations in individual samples reaching levels up to 77.5 mg/kg FB_1_ [4]. Yet, average FB_1_ concentrations in contaminated feed samples are markedly lower and range from 0.3 mg/kg to 2.0 mg/kg depending on the region [5].

Although FB_1_ is poorly bioavailable in all investigated species, it induces genotoxic, neurotoxic, nephrotoxic, and carcinogenic effects [3], a phenomenon also referred to as the “fumonisin paradox” [6]. Fumonisin toxicity is mainly based on an inhibition of the enzyme ceramide synthase, which causes a disruption of the sphingolipid metabolism [7]. The resulting alterations in levels of the free sphingoid bases sphinganine (Sa) and sphingosine (So) are reflected by an elevated sphinganine-to-sphingosine (Sa/So) ratio. In experimental animal models, the Sa/So ratio, determined in serum, urine, or various tissues, serves as a specific biomarker for FB_1_ exposure (biomarker of effect) [2]. In livestock, exposure to FB_1_ can cause lethal syndromes such as equine leukoencephalomalacia (ELEM) or porcine pulmonary edema (PPE) [8,9]. In addition, FB_1_ increases the susceptibility to pathogens such as *Bordetella bronchiseptica*, *Pasteurella multocida*, or porcine reproductive and respiratory syndrome virus and enhances the severity of related diseases [10]. Further important targets of FB_1_ toxicity are the immune system and the gastrointestinal tract [11,12,13]. Due to this wide range of negative effects on animal health and livestock productivity, guidance levels for fumonisins in animal feedstuffs have been implemented e.g., by the European Commission [14] or the Center for Veterinary Medicine of the Food and Drug Administration [15]. Recommended maximum FB_1_ concentrations in swine and poultry feed vary from 5–10 mg/kg and 20–50 mg/kg, respectively. However, recent findings underline the potential health risks deriving also from dietary FB_1_ concentrations below such guideline levels [16,17].

Since fumonisins have a significant economic impact [18], different strategies have been developed to reduce FB_1_ levels in cereal crops. Application of good agricultural practices, such as plant breeding, crop rotation, tillage or adequate harvesting time can help to control fumonisin contamination [19]. Still, these preventive measures can only reduce, but not completely eliminate FB_1_ in commodities. Consequently, post-harvest techniques are indispensable, which include chemical and physical decontamination methods and biological inactivation. In general, mycotoxins are highly stable to physical and chemical treatments [20]. Since physical and chemical decontamination techniques are cost-intensive and may result in derivatives with unknown toxicity [21], the deactivation of mycotoxins in the gastrointestinal tract of animals via adsorption or biotransformation is often more applicable in practice.

**Figure 1:**
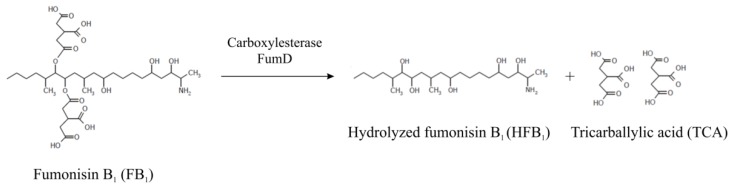
Biotransformation of FB_1_ to HFB_1_ mediated by the fumonisin carboxylesterase FumD.

Mycotoxin adsorption is based on the ability of a feed additive to bind the toxin resulting in a reduction of its bioavailability and subsequent elimination via feces. The binder-toxin complex must be stable during the complete digestion process, while the unspecific adsorption of nutrients and minerals in the feed has to be avoided. The efficacy of a binding material depends on the physical properties and chemical structure of the binder and the target mycotoxin [22]. In general, high adsorption rates have been reported *in vitro* as well as *in vivo* for aflatoxins but not for other mycotoxins, such as fumonisins, zearalenone, and trichothecenes [23]. An alternative approach for the elimination of mycotoxins *in vivo* is the biotransformation and resulting degradation of mycotoxins into non-toxic metabolites by microorganisms or purified enzymes. Several microorganisms with the capability to degrade mycotoxins have been isolated from soil, gut microbiota, or rumen fluid [24]. Previously, our group isolated the fumonisin-degrading bacterium *Sphingopyxis* sp. MTA144 from soil, elucidated the respective catabolic pathway and identified the gene cluster encoding enzymes for FB_1_ degradation [25,26,27]. Most prominently, FumD, a type-B carboxylesterase, was found to catalyze detoxification of FB_1_ by hydrolysis of both tricarballylic acid (TCA) side chains (Figure 1). The resulting compound, hydrolyzed fumonisin B_1_ (HFB_1_), has been demonstrated to have a greatly reduced toxicity compared to FB_1_ in rodents and pigs [28,29,30,31].

This article describes a set of three consecutive experiments which were performed to evaluate the potential of the fumonisin carboxylesterase FumD to detoxify FB_1_ in turkeys and pigs. First, an *ex vivo* experiment was performed to investigate the activity of FumD under digestive conditions. To this end, intestinal contents of the pig small intestine were spiked with FB_1_, incubated with or without the addition of FumD and subsequently analyzed for FB_1_ and HFB_1_. Thereafter, two feeding trials were performed to test the efficacy of the commercial application of FumD (FUM*zyme*). Turkeys and pigs were exposed to diets containing fumonisin concentrations below the recommended guideline levels for 14 and 42 days, respectively. The effect of FUM*zyme* was assessed by measuring specific fumonisin biomarkers in excreta (FB_1_, HFB_1_, partially hydrolyzed fumonisins pHFB_1_a and pHFB_1_b,) and serum (Sa/So ratio) of animals.

## 2. Results and Discussion

### 2.1. Enzyme Activity under Intestinal Conditions (Ex Vivo Model)

Several factors like pH-value or temperature can have a significant influence on the activity of enzymes. Hence, it was important to evaluate the principal capability of FumD to cleave FB_1_ under digestive conditions prior to *in vivo* efficacy tests. In a straightforward approach, different parts of the pig intestine (duodenum, jejunum) were dissected and intestinal contents were spiked with FB_1_. Subsequently, samples were treated with pure water (FB) or water containing FumD (FB+FumD). Levels of FB_1_ and HFB_1_ in the intestinal contents were monitored over a 24 h incubation period.

**Figure 2:**
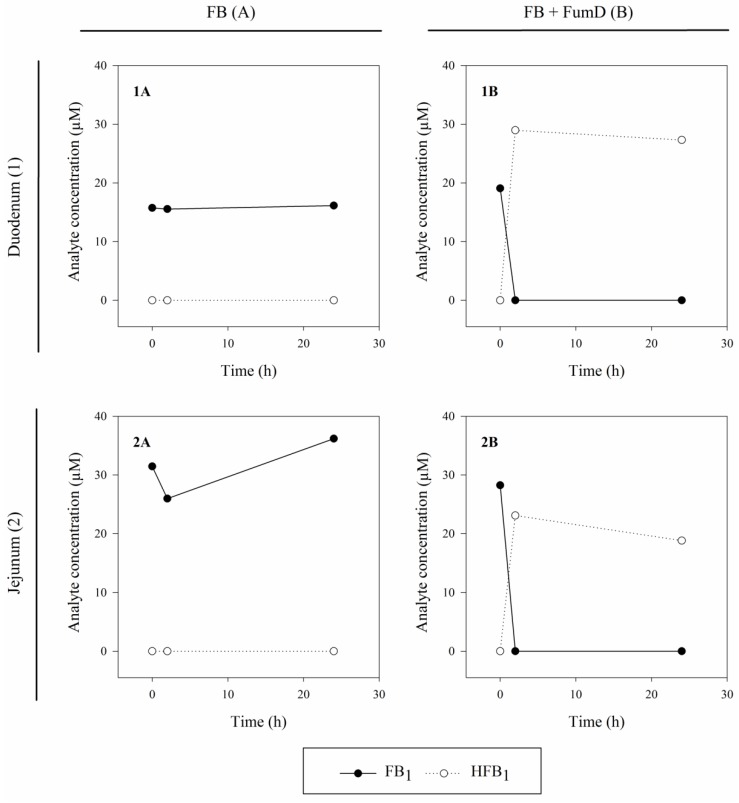
Activity of FumD under digestive conditions (*ex vivo* pig model). Concentrations of FB_1_ and HFB_1_ (µM) were determined in intestinal contents of duodenum (1) and jejunum (2) previously spiked with 5 mL of an FB_1_ solution (83.1 µM, aiming for final concentration of 13.9 µM in intestinal contents) and additionally treated with 5 mL of pure water (FB (**A**)) or 5 mL of water containing FumD (1.2 U/mL, aiming for final concentration of 0.2 U/mL in intestinal contents, FB+FumD (**B**)). Samples were collected prior to (0 h) and after 2 h and 24 h of incubation at 39 °C, respectively.

As can be deduced from Figure 2, the sole addition of water did not induce degradation of FB_1_. Toxin levels remained in the added concentration range with only slight variations in jejunum (Figure 2, inlays 1A and 2A). Toxin amounts applied in this experiment (5 mL of 60 µg/mL FB_1_ solution, 83.1 µM) were aimed at a final concentration of 10 µg/mL FB_1_ (10 µg/mL, 13.9 µM) in the individual gut segments and selected based on an estimated volume of the intestinal contents of 25 mL. However, the content of the parts dissected from the jejunum was overestimated. As a consequence, respective FB_1_ concentrations were approximately two-fold higher compared to the FB_1_ levels measured in duodenal samples. Independent of the time point and the gut segment, HFB_1_ could not be detected in FB samples (Figure 2, inlays 1A and 2A). By contrast, addition of FumD (5 mL of 1.2 U/mL FumD solution) resulted in a decrease in FB_1_ concentrations within 2 h. The degradation of FB_1_ in the intestinal samples was accompanied by an increase in HFB_1_ levels (Figure 2, inlays 1B and 2B). Partially-hydrolyzed fumonisins (pHFB_1_a, pHFB_1_b), the molecules resulting from removal of one of the two tricarballylic side chains, were not detected.

Despite the limited extent of this *ex vivo* experiment, obtained results showed the potential of FumD to degrade FB_1_ to HFB_1_ under digestive conditions. Rapid detoxification of FB_1_ in proximal parts of the intestine is of particular importance for the prevention of adverse health effects of fumonisins, mainly for two reasons. First, although no studies are currently available that elucidate the exact location or the cellular mechanism of FB_1_ uptake from the gut, it is reasonable to assume that an early degradation of FB_1_ reduces the total amount of absorbed toxin, thereby helping to prevent systemic toxicosis. Second, FB_1_ not only exhibits systemic effects in animals, but also significantly impacts gut health. For instance, this mycotoxin has been demonstrated to impair gut morphology [31,32], reduce the intestinal barrier function [33,34], alter the local immune response [17,35] and to induce the expression of stress proteins, especially in the colon [36]. Moreover, FB_1_ negatively affected the balance of the gut microbiota in pigs [37] and predisposed the intestinal colonization of a pathogenic *Escherichia coli* strain [38]. By contrast, reports on the intestinal effects of HFB_1_ are rare. Grenier *et al.* [31] observed no significant influence of HFB_1_ on the intestinal lesion score or the villi morphometry in piglets exposed to 2.8 µmol HFB_1_/kg b.w./day for two weeks. Compared to its parent toxin, HFB_1_ only slightly modified the expression of cytokines in the gastrointestinal tract. Thus, the application of FumD could represent a useful approach to counteract toxic effects of FB_1_ on the intestine [39].

### 2.2. Efficacy of FUMzyme in Turkeys

To evaluate the efficacy of the commercial application of FumD (FUM*zyme*) *in vivo*, a feeding trial with turkeys was performed. Since turkeys are more sensitive to FB_1_ exposure than poultry (summarized by Benlasher *et al.* [40]), this species was chosen as the avian model for our experiment. Turkeys were fed basal diet (CON), fumonisin-contaminated diet (15 mg/kg FB_1_+FB_2_; FB), or a fumonisin-contaminated diet supplemented with FUM*zyme* (15 mg/kg FB_1_+FB_2_, 15 U/kg FUM*zyme*; FB+FUM*zyme*) for 14 days *ad libitum*. Potential degradation of FB_1_ was investigated by analyzing excreta for FB_1_, pHFB_1_a, pHFB_1_b, and HFB_1_ by LC-MS/MS. Results for the different sampling time points (day 7, day 14) are listed in Table 1.

As expected, none of the investigated analytes was detected in excreta of the CON group, nor in the FB and FB+FUM*zyme* group prior to FB_1_ exposure (day 0). By contrast, significant levels of FB_1_ as well as minor levels of pHBF1a, pHFB_1_b, and HFB_1_ were found in excreta of turkeys fed the fumonisin-contaminated diet. In general, FB_1_ was reported not to undergo substantial metabolism *in vivo* [3]. So far, formation of pHFB_1_a, pHFB_1_b, and HFB_1_ after FB_1_ exposure has been described in monkeys [41], pigs [42], and rats [43]. Studies on the toxicokinetics of FB_1_ in avian species are limited [44]. Although the generally poor bioavailability of this mycotoxin was confirmed also for turkeys (3.2% after single oral administration of 100 mg FB_1_/kg b.w., [45]), formation of the different FB_1_ metabolites has not been investigated so far. Thus, the current study represents the first report on the occurrence of pHFB_1_a, pHFB_1_b, and HFB_1_ in turkeys, thereby contributing to an increased knowledge on the metabolism of FB_1_ in avian species. On day 7, levels of pHFB_1_a and pHFB_1_b in excreta of the FB group were quite low (mainly ranging between the respective LOD and LOQ), while HFB_1_ concentrations ranged from 125 to 269 ng/g in the individual animals. However, compared to FB_1_ excretion (2690 ± 1470 ng/g), the formation of the mentioned metabolites was negligible. Moreover, neither pHFB_1_a, nor pHFB_1_b or HFB_1_ were detected in excreta samples of day 14. This fact may be attributed to individual and time-dependent variations in formation/excretion of pHFB_1_a, pHFB_1_b, and HFB_1_, as well as to the used sampling procedure (spot samples).

Supplementation of the fumonisin-contaminated diet with FUM*zyme* resulted in a reduction of FB_1_ levels in excreta, which became statistically significant by day 14 (*p* = 0.002). Levels of HFB_1_ in the FB+FUM*zyme* group reached maximum values of 2080 ng/g and 1700 ng/g in individual samples collected on day 7 and day 14, respectively. Compared to the FB group, this increase was significant already after seven days of treatment (*p* = 0.005). Similarly, pHFB_1_b levels were markedly elevated in the FB+FUM*zyme* group on day 7 and 14. In contrast, no relevant elevation of pHFB_1_a was observed.

In order to determine whether the degradation of FB_1_ was accompanied by a reduction of its toxicity, we assessed the influence of the different treatment diets on the sphingolipid metabolism. In turkeys, FB_1_ exposure can lead to reduced weight gain, elevated feed conversion ratio, hepatotoxicity (reflected e.g., by increased organ weights, hepatocelluar lesions), alteration of serum enzymes, or suppression of the immune system [46,47]. Yet, effects of fumonisins on performance parameters or liver weights were found to become evident only at high levels of dietary FB_1_ (50–325 mg/kg FB_1_ upwards) [48,49,50]. On the contrary, disruption of the sphingolipid metabolism was observed after chronic exposure to comparable low doses of FB_1_ [40,51]. Since an increase in the Sa/So ratio precedes other signs of toxicity, this parameter was suggested as a sensitive fumonisin biomarker in turkeys [50,51].

As shown in Table 2, Sa/So ratios of the FB group were significantly elevated compared to the animals receiving the basal diet. Notably, effects on the sphingolipid metabolism were detectable as early as 14 days after fumonisin exposure in the present study. Similarly, Tardieu *et al.* [51] found increased liver Sa/So ratios in turkeys fed diets containing 20 mg/kg FB_1_ and 10 mg/kg FB_1_ for 7 and 35 days, respectively. Based on these two studies it can be assumed that dietary fumonisin concentrations at or below recommended guideline levels [14,15] have consistent influence on the sphingolipid biosynthesis in turkeys.

By contrast, serum Sa/So levels of the FB+FUM*zyme* group did not differ significantly from values obtained in the CON group. Compared to the FB group, mean Sa/So levels were significantly reduced. Hence, the biotransformation of FB_1_ to HFB_1_ observed in the excreta samples was indeed associated with the absence of sphingolipid metabolism impairment and can be therefore regarded as a detoxification process.

### 2.3. Efficacy of FUMzyme in Pigs

To test the efficacy of FUM*zyme* in pigs, a second feeding trial was conducted. Weaned piglets received either basal feed (CON), fumonisin-contaminated feed (2 mg/kg FB_1_+FB_2_; FB), fumonisin-contaminated feed supplemented with FUM*zyme* (2 mg/kg FB_1_+FB_2_, 60 U/kg FUM*zyme*; FB+FUM*zyme*) or basal feed supplemented with FUM*zyme* (60 U/kg; FUM*zyme*) for 42 days. Different treatment diets had no significant impact on performance parameters (data not shown), whereas effects on FB_1_, pHFB_1_a, pHFB_1_b, and HFB_1_ levels in feces samples collected on day 0, 14, 28, and 42 were marked (Table 3).

Detectable amounts of FB_1_ in the CON group are a reflection of the minimal fumonisin contamination of the used basal diet (as noted in Section 3.3.2). Notably, relatively high concentrations of FB_1_ were found in feces samples of the CON group on day 14 and 42. Individual differences in FB_1_ excretion might partly account for this phenomenon. However, since similar levels of FB_1_ were also detected in feces samples of the FUM*zyme* group on day 14 and 21, an inhomogeneous distribution of FB_1_ in the applied basal feed is a more likely explanation. Although this represents a limitation of our study, fecal FB_1_ levels were still significantly increased in the FB group compared to the CON group, reaching maximum concentrations of up to 16,600 ng/g in individual piglets. Additionally, certain amounts of pHFB_1_a, pHFB_1_b, and HFB_1_ were found in the feces of FB_1_ exposed animals. Hydrolysis of FB_1_ in swine has been observed previously both *in vitro* (after incubation with caecal content) [52] and *in vivo* [42]. While in these reports a predominant formation of partially-hydrolyzed FB_1_ was proposed, we found rather similar levels of pHFB_1_a, pHFB_1_b, and HFB_1_ in feces of the FB group.

Similarly to results obtained in turkeys, supplementation of the fumonisin-contaminated diet with FUM*zyme* led to significantly reduced FB_1_ levels in feces as early as day 14. This decrease was accompanied by increased concentrations of fecal HFB_1_. Whenever comparison of means was possible (analyte concentrations in respective treatment groups > LOQ), HFB_1_ levels of the FB+FUM*zyme* group were significantly elevated compared to both the FB and CON group. As expected, metabolite patterns in feces of the FUM*zyme* and CON group were rather similar.

**Figure 3:**
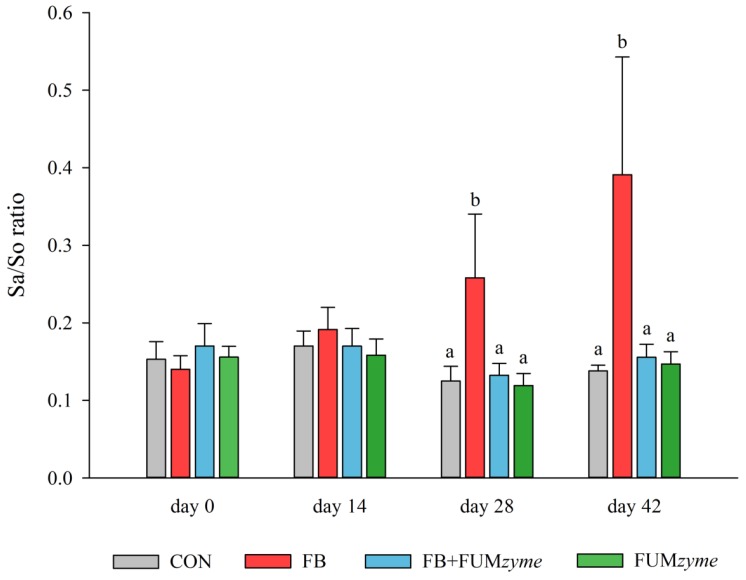
Mean serum sphinganine-to-sphingosine ratios (Sa/So) of piglets receiving either basal feed (CON), fumonisin-contaminated feed (2 mg/kg FB_1_+FB_2_; FB), fumonisin-contaminated feed supplemented with FUM*zyme* (2 mg/kg FB_1_+FB_2_, 60 U/kg FUM*zyme*; FB+FUM*zyme*), or basal feed supplemented with FUM*zyme* (60 U/kg; FUM*zyme*). Samples were collected from individual piglets (*n* = 10) prior to and after 14, 28, and 42 days of exposure to the different treatment diets, respectively. Error bars represent the standard deviation and superscript letters indicate significant differences between treatment groups (*p* < 0.05).

The effect of different treatment diets on serum Sa/So ratio is depicted in Figure 3. During the whole experiment, Sa/So ratios of the CON group remained unaffected, ranging from 0.15 ± 0.02 (day 0) to 0.14 ± 0.01 (day 42). While dietary FB_1_ had no effect on the sphingolipid metabolism after 14 days of exposure, Sa/So ratios were significantly elevated on day 28 (0.26 ± 0.08). By day 42, Sa/So ratios of the FB group had further increased (0.39 ± 0.015), reaching maximum levels of 0.76 in individual samples. On both of these sampling days, levels of Sa and So were significantly elevated compared to values obtained in the CON group. In line with Riley *et al.* [53], the increase in Sa was more prominent (5.8-fold increase on day 14, 11.4-fold increase on day 28) than the elevation of So (2.3- and 2.5-fold). Previously, feed concentrations as low as 5–6 mg/kg total fumonisins were shown to cause increased serum Sa/So ratios in pigs [39,53]. However, to the best of our knowledge the contamination level applied in the present study (2 mg/kg FB_1_+FB_2_) is the lowest one so far reported to impair the porcine sphingolipid metabolism. Since alterations in the sphingolipid biosynthesis are directly linked to adverse health effects in swine, e.g., to left-sided heart failure and onset of PPE [9], our findings underline the potential risks deriving from low dietary FB_1_ concentrations for pig health and productivity.

## 3. Experimental Section

### 3.1. Chemicals and Reagents

Acetonitrile (ACN, LC grade), methanol (MeOH, LCMS grade), formic acid, and ammonium formate were purchased from VWR International GmbH (Vienna, Austria). Water was purified using an Arium 611 VF water purification system (Sartorius, Vienna, Austria). Chemicals for preparation of enzyme buffer solution (Tris (hydroxymethyl) aminomethan, Tris; hydrochloric acid, HCl; bovine serum albumin, BSA) were obtained from Sigma Aldrich (Vienna, Austria). FB_1_ and 13C-FB_1_ mycotoxin standards were purchased from Romer Labs GmbH (Tulln, Austria). Standards of pHFB_1_a, pHFB_1_b, and HFB_1_ were produced as described in [54]. Sphingolipid standards (d-erythro-sphinganine, d-erythro-shpingosine) were from Avanti Polar Lipids (Alabaster, AL, USA).

### 3.2. Ex Vivo Model

#### 3.2.1. Preparation of Enzyme and Toxin Solutions

The fumonin caroboxylesterase FumD was produced by expression of the gene fumD in Pichia pastoris according to [25,39]. By definition, one enzyme unit (U) of FumD corresponds to the enzyme activity that releases 1 µmol tricarballylic acid per minute from 100 µM FB_1_ in 20 mM Tris-HCl buffer (adjusted to pH 8.0) containing 0.1 mg/mL BSA at 30 °C. Enzyme solution of 1.2 U/mL was prepared by dissolving FumD in water containing 0.1 mg/mL BSA. For spiking of intestinal samples, solid FB_1_ standard was dissolved in water to yield a concentration of 60 µg/mL.

#### 3.2.2. Experimental Setup

Intestines of a fattening pig (crossbred, sow: Landrace × Large White, boar: Pietrain; six months old) were collected immediately after slaughter from a local abattoir and transported on ice to the laboratory. Time between death of the animal and further preparation of intestinal samples did not exceed 30 min. From different parts of the small intestine (duodenum, jejunum), two pieces of defined length (*ca.* 10 cm) were dissected, respectively.

Subsequently, all intestinal samples were spiked with FB_1_ by sterile injection of 5 mL toxin solution (60 µg/mL FB_1_, 83.1 µM) and incubated on an orbital shaker for 1 h at 39 °C. Afterwards, 5 mL of water were injected into positive controls (one sample per gut section; FB), whereas the remaining samples were inoculated with 5 mL of enzyme solution (1.2 U/mL; FB+FumD). Applied amounts of FB_1_ and FumD were based on estimated volumes of the intestinal contents of 25 mL and should result in final concentrations of 10 µg/mL FB_1_ (13.9 µM) and 0.2 U/mL FumD in the intestinal contents. Samples of the intestinal contents were collected immediately before addition of enzyme/water (0 h) and after 2 h and 24 h of incubation (orbital shaker, 39 °C). Samples were heat-inactivated (95 °C, 5 min) and stored at −20 °C until further analysis.

#### 3.2.3. Sample Clean-Up and Analysis of FB_1_ and HFB_1_ by Liquid Chromatography-Mass Spectrometry

Samples were allowed to reach room temperature and centrifuged at 16,000× *g* for 10 min. Thereafter, 20 µL of the supernatants were evaporated to dryness under compressed air and re-dissolved in 200 µL water/acetonitrile (75/25, *v/v*; water contained 0.04% formic acid and was adjusted to pH 3.0 by addition of 6.1 mM ammonium formate buffer) containing 200 µg/L 13C-FB_1_. After final centrifugation (16,000× *g*, 10 min), sample concentrations of FB_1_, pHFB_1_a, pHFB_1_b, and HFB_1_ were determined by liquid chromatography-mass spectrometry (LC-MS) as described by Heinl *et al.* [25].

### 3.3. In Vivo Trials

#### 3.3.1. Animals and Study Design

To evaluate the efficacy of the commercial application of FumD (FUM*zyme*) *in vivo*, two consecutive feeding trials were performed. All procedures related to these experiments were carried out following the European Guidelines for the Care and Use of Animals for Research Purpose [55] and according to Austrian or Romanian law. The animal experiments were approved by the Austrian Agency for Health and Food Safety Ltd. and the Scientific Ethics Committee of the Faculty of Veterinary Medicine Timisoara (223/07032014), respectively.

First, a short-time feeding trial with turkeys was performed at the Center for Animal Nutrition (Waxenecker KEG, Mank, Austria). Female turkeys (Hybrid Converter; 10 weeks old) were obtained from a local producer. Animals were housed in groups (5 animals/pen) on wood shavings under controlled environmental conditions and a 16/8-h light/dark cycle. After an acclimatization period of seven days, 15 turkeys were individually marked and allocated to one of the following three groups (*n* = 5): negative control (CON), toxin group (FB) and product group (FB+FUM*zyme*). While animals of the FUM group received feed contaminated with 15 mg/kg FB_1_+FB_2_, the diet of the FB+FUM*zyme* group contained 15 mg/kg FB_1_+FB_2_ and 15 U/kg FUM*zyme* (Table 4). Animals had free access to feed and water for the whole duration of the trial. On days 0, 7, and 14, excreta samples were collected from all individual animals. To this end, individual animals were put in a separate sampling pen until they defecated, which did not take longer than 30 minutes. This pen was cleaned after each individual sample collection to avoid cross-contamination of feces samples. At the end of the experiment (d14), birds were killed by electric stunning and bleeding. Serum samples were collected during exsanguination. Excreta as well as serum samples were frozen at −20 °C until further analysis.

Second, a pig feeding trial was conducted at SC Pork Prod Srl (Iratos, Romania). Animals were housed in pens on scattered floor under controlled conditions and had free access to water. At weaning, 140 piglets (PIC 337; 28 days old; 70 castrated males, 70 females) were individually identified and weighted. Using a randomized block design, animals were allocated to one of four different treatment groups (*n* = 35), taking into consideration both the sex and the body weight of the animals. Piglets received either blank feed (CON), feed contaminated with 2 mg/kg FB_1_+FB_2_ (FB), feed contaminated with 2 mg/kg FB_1_+FB_2_ and substituted with 60 U/kg FUM*zyme* (FB+FUM*zyme*), or blank feed substituted with 60 U/kg FUM*zyme* (FUM*zyme*) for 42 days (Table 4). The diets were provided in dry form and *ad libitum*. On days 0, 14, 28, and 42, pigs were individually weighted. In addition, serum samples (collected from the jugular vein) and individual feces samples (collected from the rectum) were taken from ten piglets per group at each of these time points. While the number of 35 animals per group was chosen in order to match the density in industrial breeding conditions, the number of 10 animals per group for biomarker analysis was set on the basis of statistical sample size calculation. These ten piglets were randomly identified by numbers on their back at the beginning of the experiment and were then used throughout the whole experiment for sample collection. Serum and feces samples were frozen at −20 °C until further analysis.

#### 3.3.2. Feed Composition

Turkey and pig basal feed was formulated to meet species-specific requirements (Table 5) and analyzed for the presence of relevant mycotoxins, namely FB_1_ (limit of quantification, LOQ, 80 µg/kg), FB_2_ (LOQ 80 µg/kg), deoxynivalenol (LOQ 150 µg/kg), aflatoxin B1 (LOQ 0.8 µg/kg), aflatoxin B2 (LOQ 0.3 µg/kg), ochratoxin A (LOQ 0.5 µg/kg), T-2 toxin (LOQ 80 µg/kg) and HT-2 toxin (LOQ 140 µg/kg) (Romer Labs GmbH, Tulln, Austria). Mycotoxin levels exceeding the respective LOQ were only found for FB_1_ (130 µg/kg; pig basal starter feed) and deoxynivalenol (310 µg/kg; poultry basal feed).

For artificial contamination of treatment diets with fumonisins (FB groups), culture material of *F. verticillioides*, containing (10.5 g/kg FB_1_ and 4.18 g/kg FB_2_), was obtained from Romer Labs GmbH. To ensure homogeneous distribution of fumonisins within the diets, a premix was prepared using inulin. The culture material-inulin-premix was added to the basal feed at inclusion rates of 0.5% (turkey trial) and 0.05% (pig trial). Final fumonisin concentrations were verified by HPLC-MS (Romer Labs GmbH).

For the turkey trial, FumD was produced as described in 3.2.1. Each enzyme batch was separated from the biomass by centrifugation in a disk stack separator and consequently passed through micro-, ultra-, and sterile filtration to exclude any remaining cell material. Finally, the FumD concentrate was mixed with maltodextrin (10%, *w/w*), spray-dried, and further diluted with maltodextrin to obtain a FUM*zyme*-premix with 4.28 U/g. This premix was mixed into the basal feed at an inclusion rate of 0.35%. In case of the pig trial, 2 kg/t of a FUM*zyme* containing feed additive (30,000 U/kg FUM*zyme*; BIOMIN Holding GmbH, Getzersdorf, Austria) was added to the respective treatment diets. Final concentrations of active enzyme in feed were confirmed via fumonisin esterase activity assay according to [56] (BIOMIN Research Center Quality Control Lab, Tulln, Austria).

#### 3.3.3. HPLC-MS/MS Analysis of Sphingolipids

Serum sample preparation for subsequent HPLC-MS/MS determination of sphinganine (Sa) and sphingosine (So) was performed according to Grenier *et al.* [17].

Analyses were carried out on a 1290 Infinity series HPLC system (Agilent Technolgies, Waldbronn, Germany) coupled to a Triple Quad 5500 mass spectrometer (AB Sciex, Foster City, CA, USA). Chromatographic separation was achieved on a Kinetex C18 column (150 × 2.1 mm, 2.6 µm) fitted with a UHPLC C18 SecurityGuard ULTRA Cartridge (both Phenomenex, Torrace, CA, USA) at 30 °C. Eluent A consisted of MeOH/water (40/60, *v/v*) and eluent B of MeOH, both containing 0.15% formic acid. The proportion of B was increased linearly from 35% to 100% (reached at 6.5 min). After a hold-time of 3.5 min at 100% B, the column was re-equilibrated for 2.4 min at 35% B. A sample volume of 1 µL was injected into a flow of 250 µL/min.

The Triple Quad 5500 was operated in positive electrospray ionization mode, using a Turbo V ion spray source with the following settings: source temperature 550, curtain gas 40, GS1 40, GS2 40, ion spray voltage 5500, collision gas 7, dwell time 50 ms. Mass spectrometric detection was performed in selected reaction monitoring mode. In general, mass transitions chosen for analysis were as follows: *m/z* 302.3 to 60.1 (Sa quantifier; declustering potential, DP +146 V; collision energy; CE +21 eV), *m/z* 302.3 to 284.4 (Sa qualifier; DP +146 V, CE +19 eV), *m*/*z* 300.3 to 252.3 (So quantifier; DP +71 V, CV +23 eV) and *m/z* 300.3 to 282.3 (So qualifier; DP +71 V, CE +15 eV). In turkey serum samples, the mass transition of *m/z* 300.3 to 282.3 served as So quantifier, while the mass transition of m/z 302.3 to 284.4 was used as Sa quantifier.

Method validation was performed for pig as well as turkey serum samples and comprised determination of the apparent recovery (RA), the signal suppression/enhancement (SSE), the recovery of the extraction step (RE), the repeatability, limits of detection (LODs), and limits of quantification (LOQs). For this purpose, pooled serum samples of the control group were spiked with appropriate amounts of Sa and So standards prior to and after extraction in triplicate at five different concentration levels (corresponding to a working range of 1–100 ng/mL in measurement solutions for both analytes). MS data evaluation was performed using Multiquant 3.0 software (AB Sciex). RA, SSE and RE were determined as described by Sulyok *et al.* [57] and are provided in Table 6. LODs were calculated according to the equation LOD = 3s + m, where s corresponds to the standard deviation and m corresponds to the average of the calculated concentrations of 15 blank runs. Similarly, LOQs were calculated using LOQ = 10s + m. LODs of investigated analytes were between 0.6 and 1.8 ng/mL in pig and turkey serum samples, while LOQs ranged from 1.8 to 4.5 ng/mL. Repeatability, calculated as relative standard deviation of samples spiked in triplicate, was between 1% and 11%.

Serum samples of the feeding trial were worked up and analyzed in duplicate. Sa and So concentrations were determined on the basis of neat solvent calibration functions. Sa/So ratios were calculated in MS Excel (2010).

#### 3.3.4. HPLC-MS/MS Analysis of FB_1_, pHFB_1_a, pHFB_1_b, and HFB_1_

Concentrations of fumonisins in excreta of turkeys were determined by three-fold extraction of 1 g of homogenized excreta samples with 10, 10, and 5 mL of ACN/water/formic acid (74/25/1, *v/v/v*) by shaking for 30 min, 20 min, and 10 min, respectively. Aliquots of the combined supernatants after centrifugation were diluted 1+4 with extraction solvent prior to LC-MS/MS analysis according to [43,54]. Quantitative analysis was carried out on the basis of neat solvent calibration functions established in the range between 1 and 400 ng/mL. Excreta samples were worked up in duplicate and each extract was analyzed once. Excreta of pigs were analyzed accordingly, except that 300 mg of freeze-dried, homogenized sample were extracted and diluted 1+1 prior to analysis.

### 3.4. Statistical Analysis

Statistical evaluations were carried out using SPSS (Version 19.0., IBM Corp., Armonk, NY, USA) and results were considered significant at *p* < 0.05. In general, the statistical methodology included a test for normal distribution of all parameters in all groups (Kolmogorov-Smirnov test).

Normal distributed data were further analyzed by a test for homogeneity of variances (Levene’s test). If variances were homogeneous, either ANOVA followed by Tukey’s HSD test or Student´s *t*-test was performed, depending on the number of groups compared. If variances were not homogenous, data were evaluated either by Welch test with Tamhane’s T2 test as *post hoc* test or by *t*-test for inhomogeneous variances, depending on the number of groups compared. Data that were not normal distributed were further analyzed by Kruskal-Wallis test (non-parametric ANOVA) when several groups were compared or by Mann-Whitney test when only two groups were compared. Kruskal-Wallis tests were followed up by pairwise comparison (available as subfunction of the Kruskal-Wallis test in SPSS).

In groups where single analyte values were <LOD or <LOQ, half of the LOD and half of the LOQ values were used for calculation of means. At several sampling time points, FB_1_ or its metabolites were below the respective LOD und LOQ values in all individual samples of a treatment group (as indicated by “<LOD” or “<LOQ” in Table 1 and Table 3). In that case, respective data were not included in comparison of means.

## 4. Conclusions

Our data demonstrate the degradation of FB_1_ to its less toxic metabolite HFB_1_ by the use of the carboxylesterase FumD under both *ex vivo* and *in vivo* conditions. As reflected by significantly increased Sa/So ratios, dietary fumonisin concentrations below the respective recommended guideline levels caused disruption of the sphingolipid metabolism in turkeys and pigs. In contrast, feed supplementation with the commercial application of FumD (FUM*zyme*) prevented alterations of the Sa/So ratio in both species. Thus, the application of FUM*zyme* as feed additive can be regarded as effective strategy to counteract the effects of FB_1_ in turkeys and pigs.

## Figures and Tables

**Table 1:** Concentrations of FB_1_, pHFB_1_a, pHFB_1_b, and HFB_1_ (ng/g) recovered in excreta of turkeys receiving either basal feed (CON), fumonisin-contaminated feed (15 mg/kg FB_1_+FB_2_; FB) or fumonisin-contaminated feed supplemented with FUM*zyme* (15 mg/kg FB_1_+FB_2_, 15 U/kg FUM*zyme*; FB+FUM*zyme*). Samples were collected from the individual animals (*n* = 5) on day 7 and day 14. Superscript letters indicate significant differences of analytes (mean ± standard deviation (SD)) between treatment groups on a respective sampling day (*p* < 0.05).

Day	Treatment Group (*n* = 5)	FB_1_ ± SD (ng/g)	pHFB_1_a ± SD (ng/g)	pHFB_1_b ± SD (ng/g)	HFB_1_ ± SD (ng/g)
0	CON	<LOD	<LOD	<LOD	<LOD
	FB	<LOD	<LOD	<LOD	<LOD
	FB+FUM*zyme*	<LOD	<LOD	<LOD	<LOD
7	CON	<LOD	<LOD	<LOD	<LOD
	FB	2690 ± 1470	40.8 ± 32.3	<LOQ	194 ± 54.2 ^a^
	FB+FUM*zyme*	1450 ± 929	62.4 ± 46.1	308 ± 248	1340 ± 382 ^b^
14	CON	<LOD	<LOD	<LOD	<LOD
	FB	5240 ± 1930 ^a^	<LOD	<LOD	<LOD
	FB+FUM*zyme*	1190 ± 652 ^b^	<LOD	407 ± 380	1650 ± 368

FB_1_, fumonisin B_1_ (LOD 170 ng/g, LOQ 480 ng/g); pHFB_1_a, partially-hydrolyzed fumonisin B_1_a (LOD 30 ng/g, LOQ 80 ng/g); pHFB_1_b, partially-hydrolyzed fumonisin B_1_b (LOD 30 ng/g, LOQ 220 ng/g); HFB_1_, hydrolyzed fumonisin B_1_ (LOD 40 ng/g, LOQ 120 ng/g); <LOD, analyte concentration in all five individual samples below the limit of detection; <LOQ, analyte concentration in all five individual samples below the limit of quantification

**Table 2:** Serum sphinganine (Sa) and sphingosine (So) concentrations (ng/mL, mean ± standard deviation (SD)) as well as corresponding sphinganine-to-sphingosine ratios (Sa/So) of turkeys (*n* = 5) receiving basal feed (CON), fumonisin-contaminated feed (15 mg/kg FB_1_+FB_2_; FB) or fumonisin-contaminated feed supplemented with FUM*zyme* (15 mg/kg FB_1_+FB_2_, 15 U/kg FUM*zyme*; FB+FUM*zyme*) for 14 days *ad libitum*. Superscript letters indicate significant differences between treatment groups (*p* < 0.05).

Treatment Group	Sa ± SD (ng/mL)	So ± SD (ng/mL)	Sa/So ± SD
CON	6.61 ± 2.51	42.1 ± 17.4	0.16 ± 0.02 ^a^
FB	8.03 ± 1.31	34.5 ± 7.29	0.24 ± 0.02 ^b^
FB+FUM*zyme*	8.00 ± 3.49	41.4 ± 17.4	0.19 ± 0.02 ^a^

**Table 3:** Concentrations of FB_1_, pHFB_1_a, pHFB_1_b, and HFB_1_ (ng/g) recovered in feces of swine receiving either basal feed (CON), fumonisin-contaminated feed (2 mg/kg FB_1_+FB_2_; FB), fumonisin-contaminated feed supplemented with FUM*zyme* (2 mg/kg FB_1_+FB_2_, 60 U/kg FUM*zyme*; FB+FUM*zyme*), or basal feed supplemented with FUM*zyme* (60 U/kg; FUM*zyme*). Samples were collected from individual piglets (*n* = 10) prior to (day 0) and after 14, 28, and 42 days of exposure to the different treatment diets, respectively. Superscript letters indicate significant differences of analytes (mean ± standard deviation (SD)) between treatment groups on respective sampling day (*p* < 0.05).

Day	Treatment Group (*n* = 10)	FB_1_ ± SD (ng/g)	pHFB_1_a ± SD (ng/g)	pHFB_1_b ± SD (ng/g)	HFB_1_ ± SD (ng/g)
0	CON	<LOQ	<LOQ	<LOQ	<LOQ
	FB	<LOQ	<LOQ	<LOQ	184 ± 285 ^a^
	FB+FUM*zyme*	<LOQ	<LOQ	32.5 ± 17.2	<LOQ
	FUM*zyme*	<LOQ	<LOD	<LOQ	545 ± 548 ^b^
14	CON	2350 ± 1960 ^a^	314 ± 174 ^a^	366 ± 221 ^a^	355 ± 190 ^a^
	FB	6870 ± 815 ^b^	275 ± 153 ^a^	244 ± 177 ^a^	305 ± 225 ^a^
	FB+FUM*zyme*	1980 ± 394 ^a^	844 ± 223 ^b^	929 ± 246 ^b^	1820 ± 269 ^b^
	FUM*zyme*	1580 ± 609 ^a^	< LOQ	142 ± 41.8 ^a^	231 ± 72.1 ^a^
28	CON	<LOQ	<LOQ	<LOQ	<LOQ
	FB	11,900 ± 1300 ^a^	<LOQ	106 ± 26.5 ^a^	<LOQ
	FB+FUM*zyme*	2020 ± 442 ^b^	689 ± 201	703 ± 213 ^b^	1510 ± 212
	FUM*zyme*	<LOQ	<LOQ	122 ± 30.9 ^a^	<LOQ
42	CON (*n* = 9)	3170 ± 235 ^a^	<LOQ	256 ± 48.2 ^a^	<LOQ
	FB	14,900 ± 860 ^b^	252 ± 95.9 ^a^	326 ± 40.5 ^b^	349 ± 298 ^a^
	FB+FUM*zyme*	5650 ± 1390 ^c^	1170 ± 113 ^b^	983 ± 104 ^c^	1820 ± 293 ^b^
	FUM*zyme*	549 ± 322 ^d^	<LOQ	175 ± 26.5 ^d^	321 ± 153 ^a^

FB_1_, fumonisin B_1_ (LOD 56.0 ng/g, LOQ 560 ng/g); pHFB_1_a, partially-hydrolyzed fumonisin B_1_a (LOD 41.6 ng/g, LOQ 413 ng/g); pHFB_1_b, partially-hydrolyzed fumonisin B_1_b (LOD 9.6 ng/g, LOQ 51.2 ng/g); HFB_1_, hydrolyzed fumonisin B_1_ (LOD 83.2 ng/g, LOQ 416 ng/g); <LOD, analyte concentration in all ten individual samples below the limit of detection; <LOQ, analyte concentration in all ten individual samples below the limit of detection or below the limit of quantification.

**Table 4:** Concentrations of fumonsins and FUM*zyme* in different treatment diets of the turkey and pig feeding trial, respectively.

Trial	Treatment Group	∑ FB_1_+FB_2_ (mg/kg)	FUM*zyme* (U/kg)
Turkey	CON	-	-
	FB	15	-
	FB+FUM*zyme*	15	15
Pig	CON	-	-
	FB	2	-
	FB+FUM*zyme*	2	60
	FUM*zyme*	-	60

**Table 5:** Composition of basal diets used in the turkey and pig feeding trial.

Ingredient (%)	Turkey	Pig
Corn	44.2	42.3
Soybean meal	31.0	24.0
Wheat	15.0	20.0
Whey powder	-	4.5
Vegetable fat	-	1.5
Vegetable protein	-	1.1
Sunflower/palm kernel oil	3.3	1.9
Pumpkin seed cake	0.7	-
Lignocellulose	0.6	-
Calcium carbonate	1.9	0.8
Calcium phosphate	1.8	1.4
Natrium carbonate	0.3	-
Natrium chloride	0.2	0.2
Magnesium phosphate	0.1	-
Potassium diformiate		0.1
Lysine	0.3	0.6
Methionine	0.2	0.2
Threonine	0.1	0.2
Tryptophan	-	0.1
Valine	-	0.1
Vitamin/mineral-premix	0.3 ^1^	1 ^2^
Analyzed Composition ^3^		
Crude protein (g)	227	193
Crude fibre (g)	36	27
Starch (g)	441	515
Metabolizable energy (MJ)	14.2	16.2

^1^ Main ingredients and final concentrations in basal diet: vitamin A (14.1 MIU/kg), vitamin D3 (4.9 MIU/kg), vitamin E (100 mg/kg), vitamin C (87 mg/kg), vitamin B1 (4.9 mg/kg), vitamin K3 (3.5 mg/kg), iron (110 mg/kg), copper (24 mg/kg), zinc (72 mg/kg). ^2^ Main ingredients and final concentrations in basal diet: vitamin A (16875 IU/kg), vitamin D3 (2000 IU/kg), vitamin E (253.75 mg/kg), vitamin C (63.5 mg/kg), vitamin B1 (4 mg/kg), vitamin K3 (4.37 mg/kg), iron (151.12 mg/kg), copper (170.0 mg/kg), zinc (126.75 mg/kg). ^3^ Corresponding to 1000 g dry matter/kg.

**Table 6:** Method performance parameters for sphingoid bases sphinganine (Sa) and sphinogosine (So) in turkey and pig serum.

Matrix	Analyte	*R*_A_^a^ ± RSD (%)	SSE ^b^ ± RSD (%)	*R*_E_^c^ ± RSD (%)
Turkey serum	Sa	93.4 ± 1.1	98.2 ± 2.7	95.2 ± 2.9
	So	90.6 ± 0.5	93.4 ± 4.1	97.3 ± 4.5
Pig serum	Sa	82.5 ± 0.5	96.7 ± 7.4	85.6 ± 7.7
	So	79.9 ± 5.7	94.2 ± 5.9	85.2 ± 10.5

^a^ Apparent recovery. ^b^ Signal suppression/enhancement. ^c^ Extraction recovery.

## Abbreviations

The following abbreviations are used in this manuscript:

FB_1_	fumonisin B_1_
FB_2_	fumonisin B_2_
FB_3_	fumonisin B_3_
HFB_1_	hydrolyzed fumonisin B_1_
pHFB_1_a	partially hydrolyzed fumonisin B_1_a
pHFB_1_b	partially hydrolyzed fumonisin B_1_b
HPLC-MS/MS	high performance liquid chromatography-tandem mass spectrometry
LOD	limit of detection
LOQ	limit of quantification
